# Nitrogen Starvation Acclimation in *Synechococcus elongatus*: Redox-Control and the Role of Nitrate Reduction as an Electron Sink

**DOI:** 10.3390/life5010888

**Published:** 2015-03-13

**Authors:** Alexander Klotz, Edgar Reinhold, Sofía Doello, Karl Forchhammer

**Affiliations:** 1 Interfakultäres Institut für Mikrobiologie und Infektionsmedizin der Eberhard-Karls-Universität Tübingen, Auf der Morgenstelle 28, 72076 Tübingen, Germany; E-Mails: alexander.klotz@uni-tuebingen.de (A.K.); no.ed@web.de (E.R.); sofia.doello@gmail.com (S.D.); 2 Department of Molecular Biosciences and Bioengineering, University of Hawaii at Manoa, Honolulu, 96822 HI, USA

**Keywords:** *Synechococcus elongatus* PCC 7942, *nblA*, *glnB*, NtcA, nitrogen starvation response, l-methionine-sulfoximine, glutamine synthetase

## Abstract

Nitrogen starvation acclimation in non-diazotrophic cyanobacteria is characterized by a process termed chlorosis, where the light harvesting pigments are degraded and the cells gradually tune down photosynthetic and metabolic activities. The chlorosis response is governed by a complex and poorly understood regulatory network, which converges at the expression of the *nblA* gene, the triggering factor for phycobiliprotein degradation. This study established a method that allows uncoupling metabolic and redox-signals involved in nitrogen-starvation acclimation. Inhibition of glutamine synthetase (GS) by a precise dosage of l-methionine-sulfoximine (MSX) mimics the metabolic situation of nitrogen starvation. Addition of nitrate to such MSX-inhibited cells eliminates the associated redox-stress by enabling electron flow towards nitrate/nitrite reduction and thereby, prevents the induction of *nblA* expression and the associated chlorosis response. This study demonstrates that nitrogen starvation is perceived not only through metabolic signals, but requires a redox signal indicating over-reduction of PSI-reduced electron acceptors. It further establishes a cryptic role of nitrate/nitrite reductases as electron sinks to balance conditions of over-reduction.

## 1. Introduction

Cyanobacteria are photoautotrophic bacteria with minimal nutritional requirements. Nevertheless, the growth of widely distributed non-diazotrophic cyanobacteria is limited in many natural habitats by shortage of combined nitrogen sources [1]. To study the acclimation to nitrogen deprivation, the unicellular, nondiazotrophic strain *Synechococcus elongatus* PCC 7942, from now on referred to as *S. elongatus*, has been used as a model for molecular and genetic investigations [2]. Nitrogen starvation causes bleaching of the *S. elongatus* cells, a process termed chlorosis [3,4]. The visible bleaching of the photosynthetic pigments is caused by an ordered proteolytic degradation of the photosynthetic machinery [5,6,7,8]. Nitrogen-starvation induced chlorosis does not lead to cell death, but during prolonged nitrogen starvation, the cells gradually differentiate into a resting state, in which the cells remain viable for months [7,8]. The first phase of chlorosis, which initiates already after a few hours following nitrogen deprivation, is defined by the degradation of the phycobilisomes, the major light harvesting antenna [4,7]. Phycobilisomes are large protein complexes, consisting of pigmented phycobiliproteins and non-pigmented linker proteins. The degradation of phycobilisomes is mediated by NblA, a Clp protease-associated adapter protein [5,6]. The respective gene, *nblA*, was identified by screening mutants, which show a non-bleaching (nbl) phenotype [5]. Similar screens identified other *nbl* genes involved in the regulation of *nblA* expression [9,10,11]. Expression of the *nblA* gene is the trigger that initiates phycobilisome degradation and therefore, its expression is tightly controlled. The histidine kinase NblS and putative response regulator NblR were candidates of a two-component signal system that controls *nblA* expression, since mutants in both genes confer a non-bleaching phenotype [10]. However, the regulatory system involved in *nblA* expression is far more complex. NblR was shown to be a phosphorylation-independent (one component) response regulator [12,13] and not to interact with NblS [14,15,16]. On the other hand, NblS, a sensor kinase, which is highly conserved in cyanobacteria and also occurs in non-green eukaryotic algae [17] was shown to be implicated in numerous cellular processes, such as osmoregulation, oxidative stress, high light adaptation or cold-shock and has been named also Hik33, DspA, Dfr, or Ycf26 [18,19,20]. NblS interacts at least with two different response regulators of the OmpR family, RpaB and SrrA [15,21], and the phosphor-transfer reaction is further modulated by SipA, a NblS-binding protein that enhances the autophosphorylation activity and phosphotransfer reaction [14,22]. Response regulator RpaB has been shown to be a key factor in the regulation of high-light regulated genes mediated by the HLR1 motif, to which RpaB binds [23,24,25]. In its phosphorylated form, RpaB binds and represses expression of the *nblA* gene [26] and upon dephosphorylation, *nblA* expression is derepressed, leading to phycobiliprotein proteolysis.

In *S. elongatus*, an additional regulatory protein, NblR, a response-regulator homologue, is required for *nblA* derepression, however, its function remains enigmatic. To add additional complexity to the expression of *nblA*, the promoter of *nblA* is further regulated by the nitrogen-control (ntc) transcription factor NtcA [27,28]). NtcA controls almost all genes responding to nitrogen and its activity is activated by binding of 2-oxoglutarate and by PipX, a presumptive transcriptional co-activator [29,30,31]. In summary, it seems that *nblA* expression is at least under dual control: the Nbl-System (involving NblS, RpaB; NblR) appears to be required for the principal derepression of *nblA*, since mutants in these genes are impaired in bleaching under any conditions. By contrast, the NtcA mutant shows a partial non-bleaching phenotype only under conditions of nitrogen starvation [32]. The physiological signal that is sensed by the Nbl-regulatory system, when the cells experience nitrogen starvation and that leads to *nblA* derepression remains unclear. Recent studies imply that also the synthesis of glycogen is involved in the starvation-induced chlorosis process. Non-diazotrophic wild-type cyanobacteria respond to nitrogen starvation by a massive accumulation of the intracellular glycogen reserves [33,34]. However, mutants with defects in essential genes for glycogen synthesis cannot survive nitrogen starvation, they do not bleach and excrete TCA-cycle intermediates such as succinate, fumarate and 2-oxoglutarate [34,35]. Thus far, the regulatory link between glycogen synthesis and induction of the Nbl-system is unsolved.

Nitrogen starvation can be mimicked by preventing the assimilation of ammonium by glutamine synthetase (GS) using the GS-specific inhibitor l-methionine-sulfoximine (MSX). Previous studies showed that treatment with MSX, which strongly activates nitrogen-starvation responses did not lead to bleaching, but arrested the chlorosis response [32]. This observation raised the question: at which point did MSX interfere with chlorosis? This study shows that at sub-toxic concentrations, MSX indeed induces *nblA* expression and chlorosis. However, the addition of nitrate prevents this process. Under these conditions, nitrate reduction acts as an electron sink, relieving over-reduction of the cells, caused by inhibition of nitrogen assimilation. Therefore, MSX, when used in a narrow concentration window, is a valuable tool to distinguish between metabolic and redox signals involved in starvation acclimation of cyanobacteria.

## 2. Experimental Section

### 2.1. Strains and Growth Conditions

*Synechococcus elongatus* strain PCC7942 and following *S. elongatus* strains carrying *luxAB* reporter fusions were used in this study: *Synechococcus*-WT-C103 (*nblA::luxAB*) [36] and *Synechococcus*-FAM2 (*glnB::luxAB* without the constitutive σ^70^ binding site) [37] from now on referred to as WT-C 103 and FAM2. The strains containing the *luxAB*-reporter constructs were maintained in the presence of 5 µg/mL chloramphenicol.

Cells were grown in liquid BG_11_ medium at 30 °C as described previously [7]. When ammonium was used as nitrogen source, the nitrate salt from BG_11_ was replaced by 5 mM NH_4_Cl and the medium was buffered to pH 8.0 with 5 mM HEPES. BG_11_ without any nitrogen source is named BG_0_. Illumination was provided with standard fluorescent lamps (Osram daylight) at a photosynthetic photon flux density (PPDF) of 50–60 µmol photons m^−2^ s^−1^ which resulted in generation times of 15–16 h. Nitrogen step down was initiated by filtration on PVDF-membranes and resuspension of the cells in medium deprived of a combined nitrogen source, as described previously [10]. In the experimental cultures, chloramphenicol was omitted from the medium, since it turned out that chloramphenicol interfered with the luciferase read out.

### 2.2. Determination of Luciferase Activity

To determine the bioluminescence from the reporter strains, 1 ml of cell culture, adjusted to an optical density (OD_750_) of 0.45, was supplemented with decanal to a final concentration of 0.25 mM from a 50 mM stock solution made up in 10% (v/v) DMSO in H_2_O. Light emission by bioluminescence was recorded in a luminometer (Lumat LB9501, Berthold, Pforzheim, Germany). Bioluminescence values are given as relative light units (RLU), with 1 RLU corresponding to approximately 10 light impulses per second.

### 2.3. Measurement of Glutamine Synthetase (GS) Activity

Glutamine synthetase activities (transferase assay) from mid-exponential cultures were measured in permeabilized cells as described [38].

### 2.4. Photosynthetic Oxygen Evolution Measurement

As a measure for photosynthetic activity, the oxygen formation was determined with an oxygraph (Hansatech instruments RS232, Norfolk, UK). Two milliliters of exponentially growing WT-C 103 cells with an OD_750_ of 1.4 propagated in BG_11_ under standard conditions were transferred into the measurement chamber. Oxygen formation in darkness and light (35 µmol photons m^−2^ s^−1^) was measured. NH_4_Cl was added to a final concentration of 25 mM and the effect on oxygen formation was determined.

### 2.5. PAM Fluorometry

PSII activity was analyzed *in vivo* with a WATER-PAM chlorophyll fluorometer (Walz GmbH, Effeltrich, Germany), as described previously [39].

### 2.6. Determination of Colony Forming Units (CFU)

To determine the CFU per ml of a specific bacterial culture, 1 ml of culture was removed and serially diluted 10-fold in standard BG_11_ medium and of these dilutions, a volume of 100 µL was plated on BG_11_ agar plates. Five to seven days later the number of colonies growing on the plate were counted and the CFU/ml of the culture was calculated taking into consideration the dilution factor.

### 2.7. Determination of the Glycogen Content in a Culture

Glycogen was determined according to Gründel, Scheunemann, Lockau and Zilliges [33] with some modifications: 2 mL culture was harvested and the OD_750_ was measured. The cells were washed twice with sterile H_2_O and pelleted again. Then the pellet was resuspended in 400 µL 30% (w/v) KOH and incubated for 2 h at 95 °C to disrupt the cells. Then, 1.2 ml ice-cold pure ethanol (final concentration of 70%–75%) was added and incubated for 24 h at −20 °C to precipitate the glycogen. The glycogen was harvested by 10,000× g centrifugation at 4 °C for 12 min; the pellet was washed with 70% ethanol and centrifuged again at 4 °C at 10,000× g for 12 min. This step was repeated with 100% ethanol before the pellet was finally dried in a Speed-Vac at 60 °C for 40 min. To digest the glycogen, the pellet was resuspended in 1 mL of a 100 mM sodium acetate solution (pH of 4.5) and incubated for 2 h at 60 °C with ~35 U amyloglucosidase. A volume of 200 µL of this solution was added to 1 ml of a σ-toluidine reagent (6% σ-toluidine in 100% acidic acid (v/v); mature the reagent for at least 1 week) and incubated at 100 °C. Afterwards the solution was cooled for 3 min on ice and the the OD_635_ was measured. The glycogen content was calculated with a standard curve of glucose.

## 3. Results

### 3.1. MSX-Effects on Viability, nblA and glnB Expression in Ammonium Supplemented Cells

**Figure 1:**
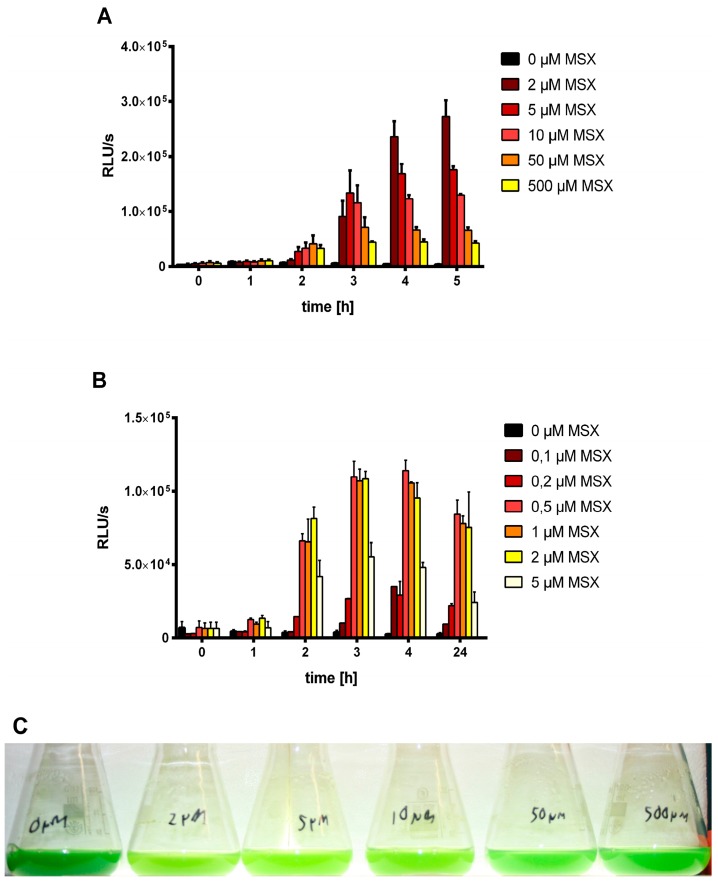
Treatment of ammonium-grown WT-C 103 cells with different concentrations of MSX (0–500 µM) (A,B) and measurement of luciferease reporter expression at time point 0, and after 1, 2, 3, 4, and 5 (**A**) as well as 24 h (**B**) of MSX treatment at concentrations indicated by color coding. (**C**) Chlorosis of WT-C 103 cells caused by treatment with MSX at the indicated concentrations for one day.

Initially, we addressed the question, why MSX, an inhibitor of GS, arrested the chlorosis response in previous studies [32]. Since chlorosis is initiated by expression of the *nblA* gene [5], we used the reporter strain WT-C 103, where the *luxAB* reporter genes are expressed under the control of the starvation-inducible *nblA* promoter. Cells that were grown in ammonium-supplemented BG_11_ medium to an optical density of 0.5 were treated with different concentrations of MSX, from 0.1 µM to 500 µM. As shown in Figure 1A,B, the effect of MSX is strongly concentration-dependent. Optimal induction was achieved with 1–2 µM MSX whereas higher concentrations inhibited the induction of *nblA*. This agrees with the visible bleaching response, shown in Figure 1C: only at high concentrations is the bleaching response inhibited.

**Figure 2:**
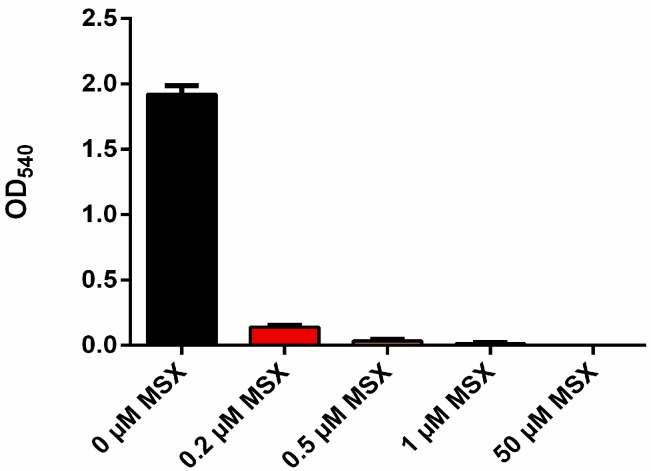
GS activity of *Synechococcus* WT-C 103 cells with different concentrations of MSX (0‑50 µM) after 4 h of incubation.

The minimal concentration of MSX needed to completely inhibit GS activity was determined to be 1 µM, whereas at lower MSX concentrations, residual GS activities were detected as shown in Figure 2. The decreasing effect of MSX on *nblA* expression below concentrations of 1 µM can, therefore, be attributed to incomplete GS inhibition.

**Figure 3:**
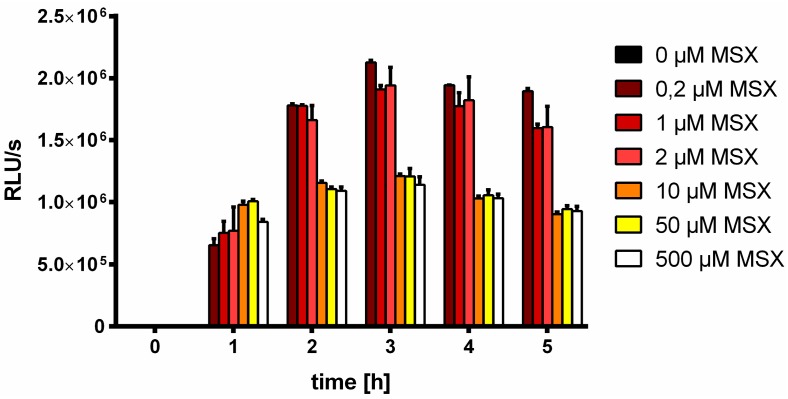
Expression of the *glnB::luxAB* reporter gene within 5 h after MSX addition at concentrations indicated using the FAM2 reporter strain and measuring luciferease activity.

To find out whether the inhibitory effect of high MSX concentrations is a global effect on gene expression, the same type of experiment with 0.2 µM up to 500 µM MSX was performed using reporter strain FAM2, which has a truncated *glnB* promoter fused to *luxAB* (Figure 3). This truncated *glnB* promoter is devoid of the constitutive σ^70^ binding site and specifically monitors NtcA-dependent gene expression [37].

Expression of the luciferase reporter in the FAM2 culture was previously shown to be specifically induced under conditions of nitrogen starvation [37]. Similar to the *nblA* the reporter strain WT-C 103, *glnB* expression was triggered by MSX concentrations in the µM range, however higher MSX concentrations had not such a drastic inhibitory effect than on the *nblA* promoter. Together, these results suggest that MSX at low concentrations indeed induces *nblA* and *glnB* promoters due to inhibition of glutamine synthetase activity and thus, metabolic conditions resembling nitrogen starvation. Higher concentrations of MSX might cause secondary effects lowering gene expression, to which *nblA* expression responds more sensitively.

**Figure 4:**
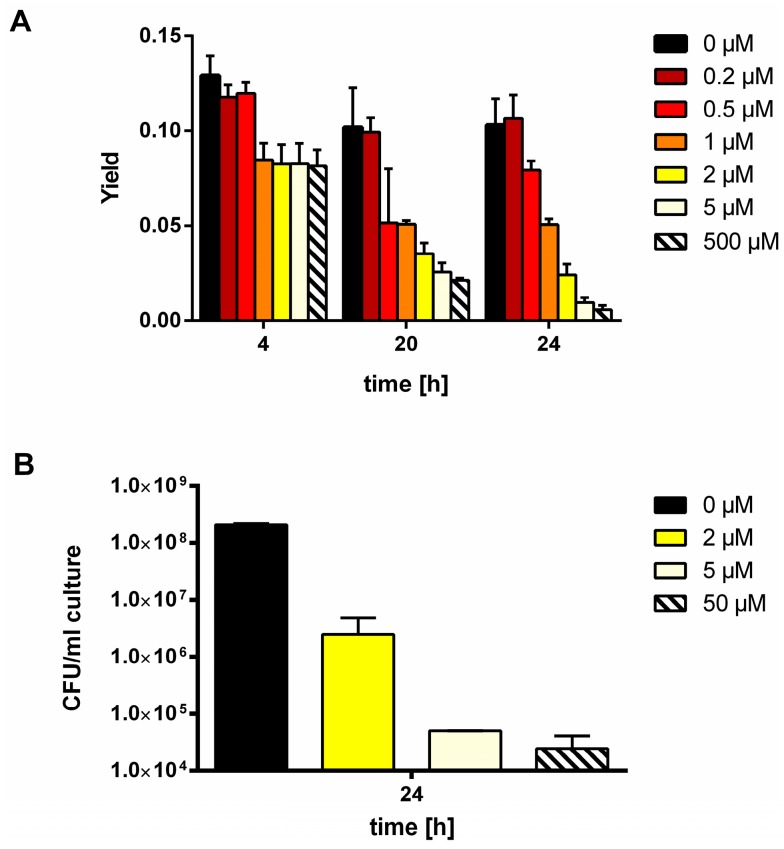
(**A**) Measurement of variable fluorescence from PSII by PAM-fluorometry after 4, 20, and 24 hours of MSX-treatment. (**B**) Determination of the colony forming units (CFU) after 24 h treatment of *S. elongatus* cells with different concentrations of MSX.

To determine, how MSX affects the viability of the cells, cultures were treated with different concentrations of MSX and at different time points; variable fluorescence from PSII was measured by PAM fluorometry as an indication of functional PSII repair. Loss of viability leads to cessation of PSII repair, and in consequence to a loss of variable PSII fluorescence [39]. As shown in Figure 4A, there is a gradual loss of variable fluorescence with increasing MSX concentrations at prolonged incubation times, whereas after 4 h of incubation, MSX, even at higher concentrations, did not severely affect PSII fluorescence. Cells, which were incubated for 24 h with 5 µM MSX or more, were not able to maintain variable fluorescence, indicating a severe loss of viability. To directly assess viability, viable counts were determined by measuring the colony forming units after culturing the cells for 24 h in presence of different concentrations of MSX. As shown in Figure 4B, 5 µM causes a severe loss of CFUs, in agreement with the vanishing variable fluorescence. By comparison, at 2 µM MSX, more than two orders of magnitude more viable cells were detected. Therefore, we decided to use MSX at 2 µM in the following experiments, a concentration, which maximally induces *nblA* expression and completely inhibits GS activity. Furthermore, we focused on the first hours of MSX treatment in order to minimize artifactual results due to toxic side effects. A further control experiment confirmed that cells treated for 5 h with 2 µM MSX are able to fully recover from this treatment (Supplementary Figure S1).

### 3.2. Nitrate Prevents MSX-Induced nblA-Expression

When the above described experiments were performed in nitrate-supplemented BG_11_ medium instead of ammonium-supplemented BG_11_, no induction of the *nblA::luxAB* reporter was observed. Therefore, we investigated the effect of nitrate in more detail. First, the induction of the *nblA::luxAB* reporter upon MSX addition was analysed in a time course experiment with cells, that were either grown in BG_11_ medium supplemented with the standard concentration of 17.7 mM sodium nitrate, or 5 mM sodium nitrite, or as before, with 5 mM ammonium chloride.

**Figure 5:**
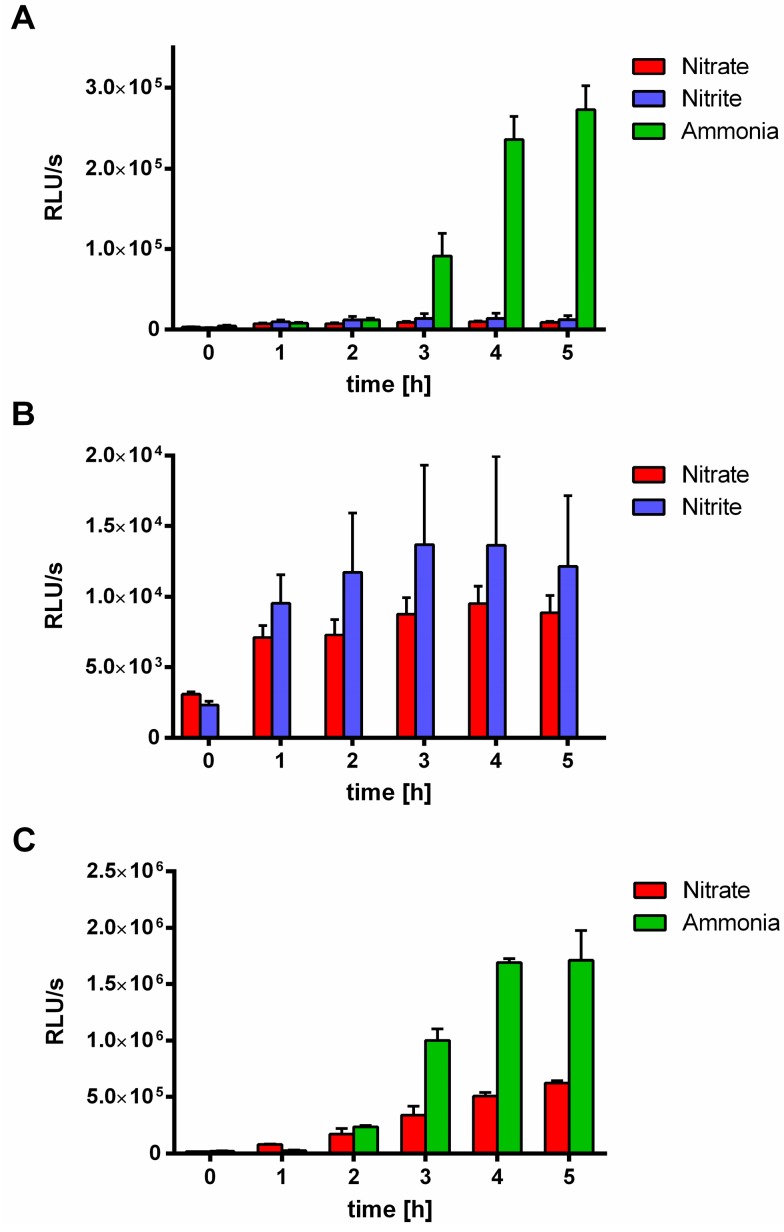
Effect of nitrate and nitrite compared to ammonia on MSX-induced *nblA::luxAB* or *glnB::luxAB* expression. (**A**) *nblA::luxAB* expression of WT-C 103 cells growing in media with different nitrogen sources within 5 h after MSX treatment (2 µM): 17.7 mM nitrate, 5 mM nitrite and 5 mM ammonia. (**B**) Highlighting of the results of *nblA::luxAB* expression of WT-C 103 in nitrate (17.7 mM) or nitrite (5 mM) containing media. (**C**) *glnB::luxAB* expression of FAM2 cells in media with nitrate (17.7 mM) and ammonia (5 mM) within 5 h after MSX treatment (2 µM).

As shown in Figure 5A, both, nitrate and nitrite almost completely prevented MSX-induced *nblA* expression. A detailed zoom into the reporter activity of nitrate- and nitrite-incubated cells showed that the inhibitory effect of nitrate is slightly stronger than that of nitrite indicated in Figure 5B. By comparison, with a reduction to about one third, nitrate did not that strongly supress the induction of the purely NtcA-dependent *glnB* promoter in the FAM-2 reporter (Figure 5C).

**Figure 6:**
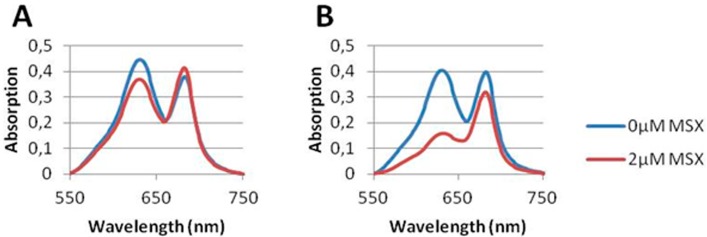
Absorbance spectra between 550 and 750 nm of *S. elongatus* PCC 7942 grown for 3 days in nitrate (**A**) or ammonia (**B**) containing medium after treatment with 2 µM MSX. To visualize the specific absorption of the pigments, the spectra were first normalized to an OD_750_ of 1, then subtracted by 1 (to set the OD_750_ to zero). Subsequently, a baseline correction was performed between OD_750_ and OD_550_ to eliminate light scattering effects.

To confirm the results shown above with pure *S. elongatus* wild-type cells, we analyzed phycobiliprotein degradation as the hallmark of chlorosis and read-out of *nblA* expression. In agreement with the inhibition of *nblA::luxAB* expression in the reporter strain, the presence of nitrate inhibited MSX-induced phycobiliprotein degradation in *S. elongatus* wild-type cells. Whereas in the presence of ammonium, 2 µM MSX treatment led to an almost complete degradation of phycobiliproteins (visible at the absorption peak at 625 nm), no phycobiliprotein degradation occurred in nitrate-supplemented medium as shown in Figure 6.

**Figure 7:**
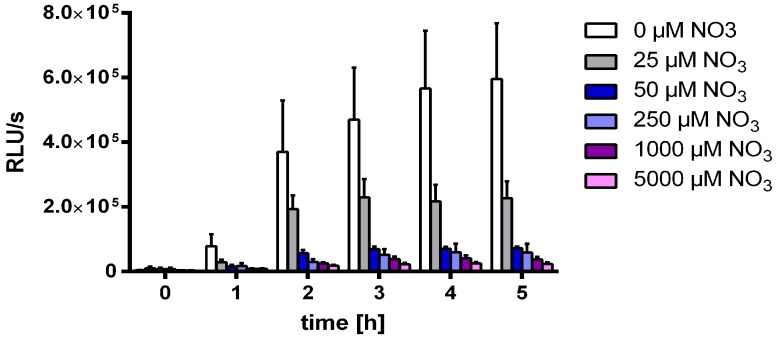
Expression of the *nblA:luxAB* reporter in strain WT-C 103 incubated in BG_0_ medium for 5 h after addition of 2 µM MSX together with different amounts of nitrate as indicated.

A possible explanation for the inhibitory effect of nitrate on MSX-induced *nblA* expression could be reduction of nitrate to ammonium via nitrate- and nitrite reductases, thereby consuming reduction equivalents produced by linear photosynthetic electron transport. In fact, MSX treated cells reduce nitrate to ammonium, which is secreted in the medium [40]. Ammonium measurement experiments confirmed that after 24 h of MSX treatment, up to 2 mM ammonium accumulated in the medium. If the electron consuming reaction of nitrate- and nitrite reductase was the explanation, one would expect a concentration-dependent effect of nitrate on *nblA* expression, since the consumption of reduction equivalents should be limited by limiting availability of substrate. To test this assumption, WT-C 103 cells growing in ammonia containing medium BG_A_ were transferred to medium containing limiting amounts of nitrate (0 µM, 25 µM, 50 µM, 250 µM, and 1000 µM). Then, 2 µM MSX was added and luminescence was measured. As shown in Figure 7, nitrate concentrations above 25 µM were sufficient to prevent *nblA* expression.

### 3.3. Efficient Photosynthetic Electron Transport is Necessary for MSX-Induced nblA Expression

**Figure 8:**
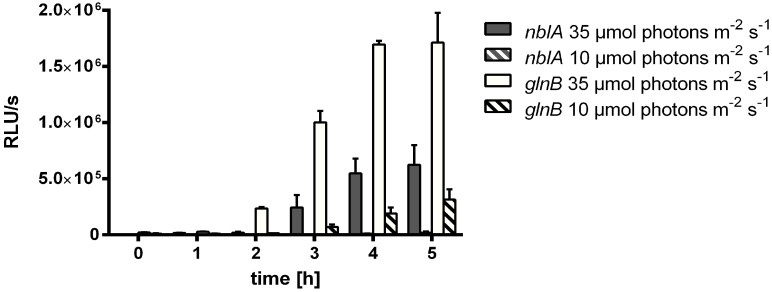
Effect of reduced PPDF on MSX-induced *nblA* and *glnB* expression. WT-C 103 and FAM2 cells were treated with 2 µM MSX prior to measurement and further propagated for 5 h under different light intensities (10 and 35 µmol photons m^−2^ s^−1^).

The clear correlation between nitrate addition and inhibition of *nblA::luxAB* suggested that the inhibitory effect of nitrate on MSX-induced *nblA* expression is indeed due to consumption of photosynthetically generated reduction equivalents through nitrate/nitrite reduction. If this assumption is correct, then one can expect a decrease of MSX-induced *nblA* expression also by lowering photosynthetic electron transport. The simplest way of reducing photosynthetic electron transport is to decrease the photosynthetic photon flux density. In fact, by lowering the PPDF from 35 to 10 µmol photons s^−1^ m^−2^ the expression of *nblA* was completely abolished, whereas the purely NtcA-dependent *glnB* promoter was still expressed, although expression was diminished to 15% residual reporter activity as shown in Figure 8.

However, one could argue that this effect could be a direct effect of light mediated through a light sensitive signalling pathway instead of a diminished photosynthetic electron transport. To rule out a direct light effect, an experiment was designed, where photosynthetic electron transport was diminished at a constant light intensity. Ammonium at higher concentrations blocks the water-splitting reaction on photosystem II, thereby lowering linear photosynthetic electron transport [39,41]. Therefore, cells that were grown in the presence of 5 mM ammonium were supplemented with additional 20 mM ammonium or not. In the presence of 25 mM ammonium, only 10% oxygen evolution was measured, compared to oxygen evolution in presence of 5 mM ammonium, as specified in Table 1.

**Table 1:** Light-dependent oxygen evolution in WT-C 103 cells in absence or presence of 25 mM ammonia. Means of triplicate measurements with standard deviation less than 5% are shown.

Conditions	Formed oxygen (nm/mL)
Darkness (0 µmol photons m^−2^ s^−1^)	0
Light (35 µmol photons m^−2^ s^−1^)	12.3
Light (35 µmol photons m^−2^ s^−1^) + 25 mM NH_4_	1.2

**Figure 9:**
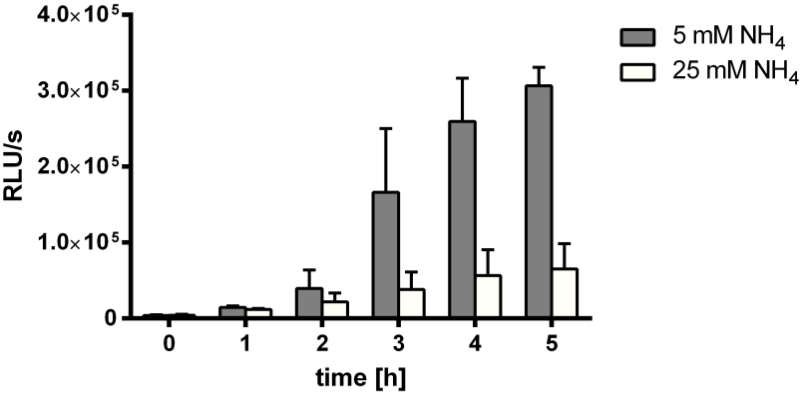
Bioluminescence measurement of *nblA* expression within 5 h after 2 µM MSX treatment and different amounts of ammonia (5 mM and 25 mM).

When the WT-C 103 cells were treated with MSX in the presence of 25 mM ammonium, a drastic reduction of *nblA* induction was observed, indicating that in fact the electron transport and not the light *per se* is required for inducing *nblA* expression shown in Figure 9. Control experiments revealed that the addition of MSX does not strongly inhibit photosynthetic oxygen evolution (Supplementary Table S1).

**Figure 10:**
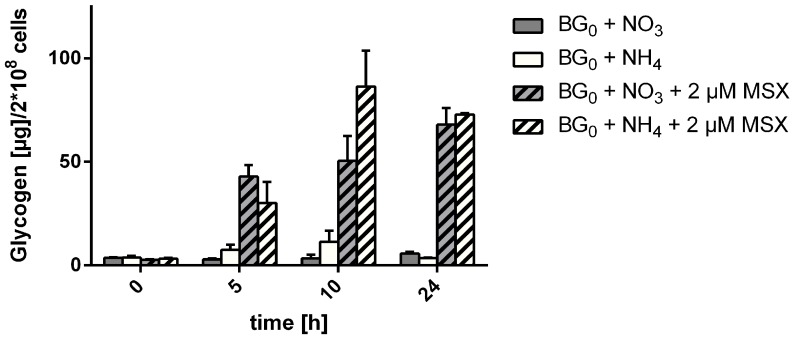
Determination of intracellular glycogen content in WT-C 103 cells incubated in BG_0_ with nitrate or ammonia with or without 2 µM MSX within 24 h.

Finally, we checked whether MSX may differentially interfere with glycogen synthesis in the presence of ammonium or nitrate, and thereby could affect *nblA* induction. Therefore, we measured the accumulation of glycogen in the presence of nitrate or ammonia, with or without addition of 2 µM MSX. As shown in Figure 10, glycogen accumulated upon MSX treatment and there was no significant difference in glycogen accumulation between nitrate and ammonium conditions after 5 and 24 h of MSX treatment. In the presence of ammonia, the glycogen maximum was already reached after 10 h, whereas the glycogen level in the presence of nitrate at 10 h was slightly lower, which might be accounted for the consumption of reducing equivalents by nitrate and nitrite reductase.

## 4. Discussion

The general nutrient-starvation response is initiated by expression of the *nblA* gene, encoding a phycobiliprotein degradation factor, which leads to chlorosis. In *S. elongatus*, *nblA* is de-repressed under various nutrient-limiting conditions, such as sulphur- or nitrogen starvation as well as under high-light stress [2,5,9,10]. Regulatory proteins controlling *nblA* expression have been identified, the NblS sensor kinase phosphorylates under non-inducing conditions the response regulator RpaB, thereby repressing *nblA* expression [14]. The nitrogen-specific transcription factor NtcA plays a role as a pathway-specific activator of *nblA* under conditons of nitrogen starvation [42]. However, the role of the putative response regulator NblR, which is required for *nblA* induction, is still enigmatic. Moreover, the signals perceived by the Nbl-regulatory system, leading to *nblA* derepression are still not understood. A recent study suggested that a critical factor in acclimation to nitrogen limitation is actually the reduction of photosynthetic electron flow through degradation of the light-harvesting antenna to avoid photo-damage [43,44]. The present study adds important information to this issue by clarifying that over-reduction of electron acceptors from photosynthetic electron transport is a necessary trigger to initiate de-repression of *nblA*. The previously observed inhibitory effect of MSX turned out to be an artifact of too-high MSX concentrations. MSX concentrations above 5 µM seem to be toxic and impair gene expression. In a narrow concentration range, high enough to completely inhibit GS activity but low enough to not damaging cell-viability within the first hours of treatment, MSX allows to uncouple metabolic and redox-signals. The inhibition of GS by MSX mimics a situation of nitrogen starvation: in the absence of ammonium, GS lacks substrate and cannot produce glutamine, which apparently is perceived in a similar manner than directly inhibiting GS activity by MSX. In agreement, this study shows that MSX in the presence of ammonium triggers the induction of the NblS/RpaB-regulated *nblA* promoter as well as the NtcA-specific *glnB* promoter. In the presence of nitrate, however, *nblA* induction is abrogated, whereas *glnB* expression is only partially affected. Nitrate is known to be a potent electron sink in cyanobacterial metabolism. In the presence of nitrate, hydrogen production, e.g., induced by sulfur-starvation is severely diminished due to consumption of reduction equivalents by nitrate and nitrite reductase [45,46]. Cyanobacterial nitrate- and nitrite reductases oxidize the photosystem I-reduced ferredoxin pool [47] and it is this effect on the redox-state that seems to be the most likely reason for the inhibitory effect of nitrate and nitrite on *nblA* expression. In MSX-treated cells with fully inhibited GS, the metabolic reactions derived from glutamine should be independent from the presence of nitrate or ammonium. Therefore, a compound of nitrogen metabolism can be ruled out as an effector molecule. A remaining alternative explanation would be a direct sensing of nitrate or nitrite in a signal transduction pathway leading to *nblA* expression. However, this explanation is very unlikely since lowering the photosynthetic electron transport by different methods in MSX-treated cells has the same effect on *nblA* expression than nitrate or nitrite. Furthermore, the fact that nitrate and nitrite are almost equally well effective argues against a direct sensing. Therefore, inhibition of *nblA* expression by nitrate or nitrite can reasonably only be attributed to a redox-signal associated with photosystem-I-reduced ferredoxin, the substrate for nitrate and nitrite-reductase. Interestingly, the solely NtcA-dependent *glnB* promoter fragment used in the FAM2-reporter is also sensitive to nitrate treatment, albeit less severe. This could indicate that activation of the NtcA factor is positively influenced by the same redox-signal that activates *nblA* expression. Redox-control of NtcA activity was previously shown to play a role in *Synechocystis* sp*.* PCC 6803 [48], but the mechanistic details were not followed up subsequently.

An important insight regarding the signals associated with the induction of chlorosis comes from the measurement of glycogen levels. It was proposed that the accumulation of glycogen may be directly linked to the induction of *nblA* expression and nitrogen chlorosis [34]. In fact, MSX-treatment triggers a similar accumulation of glycogen as observed following nitrogen starvation, with nitrogen-starved cells accumulating up to 50% glycogen [34]. Although in MSX-treated cells nitrate abrogated the expression of *nblA*, it did not prevent glycogen accumulation. This rules out that glycogen accumulation is sufficient to induce nitrogen chlorosis. Glycogen accumulation in presence of MSX can be taken as evidence that MSX does not interfere with CO_2_ fixation, the source for reduced carbon for glycogen synthesis, nor that MSX impairs photosynthetic electron transport, in agreement with oxygen evolution measurements. MSX adjusted to the right concentration acts very specifically on nitrogen assimilation and perfectly mimics a situation of nitrogen-starvation. 

Another aspect of this study is the emergence of a dual role for nitrate- and nitrite-reductase activities. Whereas these enzymes are usually referred as assimilatory nitrate- and nitrite-reductases, this study shows that under circumstances of redox-stress these enzymes are used to balance the redox-state, similar to the function of dissimilatory nitrate reduction (nitrate respiration) in heterotrophic bacteria. Cyanobacteria have evolved sophisticated mechanisms to avoid potential damage due to over-reduction of electron acceptors. The specific flavodiiron proteins (Flv1 and Flv3) have recently been shown to be responsible for safeguarding PSI under conditions of fluctuating light intensity, by oxidizing reduction equivalents directly with oxygen [49]. Under anaerobic fermentative conditions, hydrogenases have been shown to act as transient electron valves [45,46]. The present study highlights the role of nitrate/nitrite reductases as important electron sinks to avoid over-reduction of the cells. Induction of the chlorosis response appears to be used as the last resort to avoid over-reduction. The carrier of the redox-signal, that induces the Nbl-System remains to be identified. The fact that the signal is eliminated by nitrate- and nitrite reduction implies that it is, directly or indirectly, associated with PSI-reduced ferredoxin, the electron donor to nitrate- and nitrite-reductase in cyanobacteria.

## 5. Conclusions

This study established a method that allows uncoupling metabolic and redox-signals involved in nitrogen-stress acclimation. Inhibition of GS by a precise dosage of MSX brings about the metabolic situation of nitrogen starvation, and addition of nitrate eliminates the associated redox-stress. This method appears as a valuable tool for further studies to fully understand the integrated signals governing nutrient stress acclimation in cyanobacteria.

## Supplementary Materials

Supplementary materials can be accessed at: http://www.mdpi.com/2075-1729/5/1/888/s1.
