# Supplementary material for: Nitrogen Starvation Acclimation in Synechococcus elongatus: Redox-Control and the Role of Nitrate Reduction as an Electron Sink
Source: Life (Basel). 2015 Mar 13;5(1):888–904. doi: 10.3390/life5010888 (PMC4390884; doi:10.3390/life5010888)
Supplement: Supplementary file 1 [file life-05-00888-s001.pdf]

**Supplementary Materials**

**Table S1.** Oxygen evolution measurement in WT-C 103 cells incubated in $BG_0$ with nitrate or without nitrogen source supplemented with 2 µM MSX for 5h.

| Culture conditions | Mean oxygen evolution [nmol/ml/min] with standard deviation |
|---|---|
| $+NO_3$ -MSX | 10 ± 0.4 |
| $+NO_3$ +2 µM MSX | 6.2 ± 0.1 |
| $-NO_3$ -MSX | 6.8 ± 0.3 |
| $-NO_3$ +2 µM MSX | 4.8 ± 0.3 |

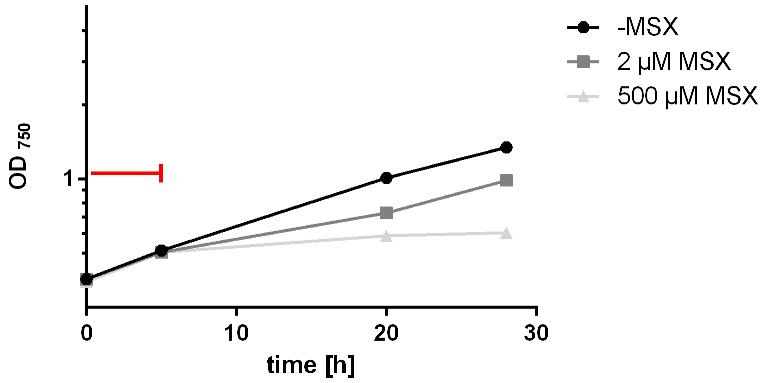


**Figure S1.** Effect of different MSX concentrations on growth of *Synechococcus* WT cells. Cells were incubated for 5 hours with MSX as indicated (red bar indicates the time of MSX incubation). Then, the cells were washed twice to completely remove MSX and afterwards, cells were resuspended in standard BG11 medium and incubated under normal growth conditions. At the indicated time points, cell density was determined by $OD_{750}$ measurement. This experiment was performed in 3 parallel biological replicates with below 5% standard deviation.
